# Supplementary material for: How charitable giving affects litigation duration? Empirical evidence from China
Source: PLoS One. 2024 Dec 30;19(12):e0316180. doi: 10.1371/journal.pone.0316180 (PMC11684597; doi:10.1371/journal.pone.0316180)
Supplement: S1 Appendix — (DOCX) [file pone.0316180.s001.docx]

**Appendix：The statutory time limit for first-instance trials in civil, administrative, criminal, and arbitration cases in China**

1. Civil Procedure Law of the People's Republic of China (2023 Amendment)

Article 164 A people's court which tries a case under the summary procedure shall complete the trial of the case within three months after the case is docketed. If an extension is needed under special circumstances, an extension of one month may be granted with the approval of the president of the court.

Article 152 A people's court shall complete the trial of a case under formal procedure within six months after the case is docketed. If an extension of the period is necessary under special circumstances, the period may be extended for six months with the approval of the president of the court; and any further extension shall be subject to the approval of the superior of the people's court.

Article 130 Where a party raises any objection to jurisdiction after a case is accepted by a people's court, the party shall file the objection with the people's court during the period of submitting a written statement of defense. The people's court shall examine the objection. If the objection is supported, the people's court shall issue a ruling to transfer the case to the people's court having jurisdiction; or if the objection is not supported, the people's court shall issue a ruling to dismiss the objection.

Article 149 Under any of the following circumstances, a court session may be postponed:

(1) A party or any other litigation participant which must appear in court fails to appear in court for justifiable reasons.

(2) A party files an unexpected request for disqualification.

(3) It is necessary to notify a new witness to appear in court, collect new evidence, conduct re-identification or resurvey, or conduct further investigation.

(4) Other circumstances requiring postponement.

Article 153 Under any of the following circumstances, an action shall be suspended:

(1) A party dies and it is necessary to wait for his or her successors to indicate whether they will participate in the action.

(2) A party loses his or her litigation competency and his or her legal representative has not been determined.

(3) A party which is a legal person or any other organization is terminated and the successors to the rights and obligations of the party have not been determined.

(4) A party is unable to participate in the action for reasons beyond the party's control.

(5) The action must depend on the results of the trial of another case which has not been concluded.

(6) Other circumstances requiring suspension.

The action shall resume after the cause of suspension is eliminated.

1. The Administrative Litigation Law of the People's Republic of China (2017 Revision)

Article 81 A people's court of first instance shall enter a judgment within six months from the day when a complaint is docketed. Any extension of the aforesaid period as needed under special circumstances shall be subject to the approval of a Higher People's Court. Where a Higher People's Court trying a case as a court of first instance needs to extend the aforesaid period, the extension shall be subject to the approval of the Supreme People's Court.

Article 83 An administrative case to which the summary procedure is applied shall be tried by a sole judge, and be closed within 45 days from the day when the complaint is docketed.

1. Criminal Procedure Law of the People's Republic of China (2018 Amendment)

Article 208 A people's court shall announce a sentence for a case of public prosecution within two months, or three months at the latest, after accepting the case. For a case with the possibility of a death penalty or a case with an incidental civil action or under any of the circumstances as set forth in Article 158 of this Law, the period of trial may be extended for three months with the approval of the people's court at the next higher level; and, if more extension is needed under special circumstances, the extension shall be reported to the Supreme People's Court for approval.

Where the jurisdiction of a people's court over a case is changed, the period of trial of the case shall be counted from the day when the people's court having jurisdiction receives the case after the change.

For a case under supplementary investigation by a people's procuratorate, a people's court shall count anew the period of trial of the case after the supplementary investigation is completed and the case is transferred to the court.

Article 212 A people's court may conduct mediation in a case of private prosecution; and the private prosecutor may voluntarily reach a settlement with the defendant or withdraw the private prosecution before a sentence is announced. Mediation shall not apply to a case as described in item (3), Article 210 of this Law.

The period for a people's court to try a case of private prosecution shall be governed by paragraph 1 or 2, Article 208 of this Law if the defendant is in custody; or a sentence shall be announced within six months after the case is accepted if the defendant is not in custody.

1. Arbitration

There are no strict statutory limits on the duration of hearings in commercial arbitration, but arbitral institutions and arbitral tribunals will endeavour to ensure that the arbitral proceedings are conducted efficiently.
