# Supplementary material for: How charitable giving affects litigation duration? Empirical evidence from China
Source: PLoS One. 2024 Dec 30;19(12):e0316180. doi: 10.1371/journal.pone.0316180 (PMC11684597; doi:10.1371/journal.pone.0316180)
Supplement: Attachment — Submitted filename: Response to Reviewers.pdf [file pone.0316180.s003.pdf]

Dear Reviewers,

I would like to express our sincere gratitude to the four reviewers for their careful review and valuable suggestions. I am honoured and encouraged to receive your review comments. Your professional scrutiny, profound insights, and insistence on academic rigour not only provided valuable guidance for our research, but also prompted me to constantly reflect and improve, and strive to make the paper even better.

I have carefully reviewed the comments and suggestions from four reviewers, and have made the necessary revisions to address each point. I believe that these changes have strengthened the paper's theoretical framework, methodological approach, and empirical findings. Below is a summary of the revisions I have made.

	**Reviewer-1**		
	Observations	Reply	indexing
1	The author provides a literature review as a very short communication, so it is strongly recommended that a more specific literature review be provided, which would be beneficial for the reader.	In accordance with your valuable suggestion, I have provided a more specific literature review in the main body of the paper. This review offers a more detailed presentation of the research findings related to charitable giving, litigation duration, and the interrelationship between these two aspects. Based on this expanded review, I have highlighted the significance and novelty of the current study. Specifically, I have organized the literature into two main sections:<br>(1)Research on the Motivations for Charitable Giving and CSR:<br>Through a systematic review and analysis of the literature, it becomes evident that existing research on the relationship between charitable giving and judicial activities primarily focuses on the early litigation risks and later litigation outcomes. However, it overlooks an in-depth examination of the litigation process itself.<br>(2)Research on External Factors Influencing Judicial Decision-Making:<br>The analysis reveals that Chinese judicial bodies, when handling cases involving listed companies, may be influenced by factors beyond the law (such as corporate charitable donations). This influence appears to affect the litigation cycle at the procedural law level.	See pages 6-11 of the Manuscript.
2	Could the author please explain more about the functions of Articles 164	(1)On the Functions of Article 164 and Article 152 of the Civil Procedure Law:<br>Articles 164 and 152 of the Civil Procedure Law	

	and 152 of the Code of Civil Procedure and what main role they play in governing litigation and the legal system in this study?"	stipulate the timeframes for the trial of civil cases under the simplified procedure and the ordinary procedure, respectively. For cases subject to the simplified procedure and ordinary procedure, the time from filing to conclusion is set as "3+1+x" and "6+6+x," meaning that, under normal circumstances, a civil lawsuit should be concluded within 4 months and 12 months, respectively.<br>(2)On the Role of Articles 164 and 152 of the Civil Procedure Law:<br>The inclusion of these articles is intended to demonstrate that the duration of litigation can be assessed and balanced by the court or the presiding judge. Naturally, such assessments are subject to various external factors that may influence this balancing process, such as the positive reputation effects generated by charitable donations, as discussed in this paper. This also reflects the theoretical exploration of a causal relationship between charitable giving and litigation duration.	
3	The conclusion is too lengthy and resembles an introduction. Please revise and rewrite it clearly. Additionally, I suggest that the author create a separate section for the conclusion, limitations, and directions for future research.	In accordance with your suggestion, I have streamlined the conclusion section and reorganized it following a logical structure: conclusion, recommendations, limitations, and directions for future research.	See pages 36-38 of the Manuscript.
4	Please, the author, carefully revise the paragraph by paragraph; I see some references are missing, so please provide the citation below and also carefully check others in your manuscript.	I have added the references that you pointed out as missing in the paper and have thoroughly reviewed and refined the other sections of the manuscript. Below are the relevant paragraphs and their corresponding reference sources as mentioned.<br>(1)Corporate reputation represents a significant intangible asset that profoundly influences consumer behavior, market direction, and societal opinion(Guan et al., 2019). [1]The events surrounding Wahaha in 2024 underscore the substantial impact corporate reputation can have	

1 Guan Kaolei, Zhang Rui. Corporate Reputation and Earnings Management: A Perspective of Effective Contracts or Rent-Seeking? [J]. Accounting Research, 2019, (01): 59-64(in Chinese).

		on stakeholders such as consumers, competitors, and investors. Consequently, numerous enterprises, particularly listed companies, are augmenting their investments in reputation assets, with charitable donations serving as a pivotal strategy(Gao et al., 2012).[2]<br>(2)The marginal contributions of this paper are fourfold. Firstly, it broadens the research scope of corporate charitable donations to the realm of judicial litigation, empirically examining the actual impact of such donations on the litigation cycle of listed companies(Fu et al., 2017).[3]<br>(3) In response to the first logical challenge, we employ Reputation Theory to elucidate the underlying mechanisms(Jean, 1996).[4]<br>(4) Reputation Theory, originating in the 19th century with economists such as Adam Smith, underscores the significance of reputation as a comprehensive public evaluation of an individual or organization's past behavior and performance in a specific social context (Smith, 1763).	
	**Reviewer-2**		
1	The regression model does not control for any fixed effects.	I have included controls for provincial and year fixed effects in the regression model. To enhance readability for the readers, I have strengthened the corresponding explanations in the model and variable settings section.	See page of 14-15the Manuscript.
2	It is not clear at what level clustered standard errors are controlled for.	The standard errors used in this paper are heteroscedasticity-robust standard errors. Since the data employed in this study consists of mixed cross-sectional data, which includes multiple distinct individuals, heteroscedasticity is commonly present. Therefore, heteroscedasticity-robust standard errors are applied. Additionally, the type of standard errors used is indicated in the manuscript.	See footnote to table 4 on page 21 of the Manuscript.
3	There is a lack of justification for the selection of IV,	The use of average donations by province, industry, and enterprise nature as an instrumental variable in this paper fulfills the criteria for	See page 24-25 of the Manuscript.

2 Gao Yongqiang, Chen Yajing, Zhang Yunjun. Corporate Reputation, Charitable Donations, and Consumer Responses [J]. Contemporary Economic Management, 2012, 34(06): 20-25(in Chinese).
3 Fu Chao, Ji Li. Litigation Risk and Corporate Charitable Donations: An Explanation from the Perspective of "Reputation Insurance" [J]. Nankai Business Review, 2017, 20(02): 108-1211 (in Chinese).
4 Tirole, Jean. “A Theory of Collective Reputations (with applications to the persistence of corruption and to firm quality).” The Review of Economic Studies 63 (1996): 1-22.

	particularly whether it meets the requirements of relevance and exogeneity.	relevance and exogeneity.Furthermore, the rationale for selecting the instrumental variables is further elaborated in the main text.	
4	The mechanism analysis only examines the impact of charitable donations on corporate reputation, but lacks justification for the complete causal chain of "charitable donations → corporate reputation → litigation efficiency." It is also reasonable to question whether there might be a competing causal relationship of "charitable donations → litigation efficiency → corporate reputation.	In response to your suggestion, I have refined the full causal chain argument of "charitable donations → corporate reputation → litigation efficiency." Specifically, I employed the Poisson regression method to capture the mapping relationship between charitable donations and reputation. Additionally, I used the same ordinary least squares (OLS) model as in the baseline regression to fit the relationship between corporate reputation and litigation duration (the supplementary regression results are presented in columns 7 and 8 of Table 7). The refined regression results support the proposed "charitable donations → corporate reputation → litigation efficiency" mechanism and reject the competing causal relationship of "charitable donations → litigation efficiency → corporate reputation."	See pages 26-27 of the Manuscript.
5	Table 11 is insufficient to support the examination of the "reputation insurance" function of charitable donations, as the argument is at least incomplete. It is not detailed whether litigation involvement risk and defendant risk differentiate between the current and lagged periods.	(1)On the Argument for Charitable Donations as "Reputation Insurance"<br>I used the charitable donations of listed companies in the current year to replace the donations in the subsequent period (Fu et al., 2017), which largely supports the "reputation insurance" function of charitable donations in contrast to existing studies. This is because corporate charitable donations are made continuously throughout the year, and according to descriptive statistics, the average litigation duration is one year. Thus, the charitable donations made by firms in the current year effectively precede the conclusion of the litigation.<br>(2)Explanation of Litigation Risk and Defendant Risk in the Current and Lagged Periods<br>There are two reasons for directly using the current period's donations and litigation risk: First, I referred to studies by Wang et al. (2008) and Lin et al. (2015), and found that in the study of litigation risk, there is limited consideration of lagged effects in academic research. Second, as explained in the first point, since the average litigation duration is one year, corporate charitable donations	

		in the current year precede the ongoing litigation in that same period. Therefore, there is no need to apply a lagged treatment.	
6	In Table 4, the two groups have observations of 1010 and 633, but the sum does not equal the total of 1710.	The reason why the number of observations in the grouped regression (1010 + 633 = 1643) is smaller than the total sample size of 1710 is that, in the variable representing party identity, in addition to the "plaintiff" and "defendant" categories, there are a small number of observations classified as "third party" or "unspecified." To enhance clarity for readers, I have included a footnote in Table 4 to provide an explanation.	See page 21 of the Manuscript.
7	In columns 2 and 4 of Table 4, the regression coefficients for law and government are insignificant, which contradicts the discussion in the main text.	The regression coefficients for law and government presented in the main text are based on columns (5) and (6) of Table 4. To avoid potential misunderstandings, I have revised the corresponding statements in the manuscript accordingly.	See pages 21-22 of the Manuscript.
8	Heterogeneity analysis shows that in the western regions of China, the regression coefficient for charitable donations is significantly positive, and there is no reasonable explanation for this result.	In the subsample analysis for the western region, the significantly positive regression coefficient of charitable donations may be attributed to the disproportionate number of cases where firms from this region are defendants. To address this, I conducted a regional heterogeneity analysis focusing on subsamples where listed companies act as plaintiffs and incorporated the corresponding regression results and interpretations into the manuscript. The results indicate that charitable donations by listed companies in the northeastern region exhibit the most significant effect in reducing litigation duration. Furthermore, when firms in the eastern, central, and northeastern regions serve as plaintiffs, the negative impact of charitable donations on litigation duration is more pronounced, whereas this effect is weaker in the western region.	See pages 29-31 of the Manuscript.
	**Reviewer-3**		
1	Please note that this article, which is very structured and persuasive, should not	Thank you for your recognition of this study and for your valuable feedback. Based on your comments, I have made the following responses and revisions:	

	be published twice, particularly in journals from China, if you agree to be accepted.<br>Though using the term "efficiency" in the title, I see no strong connection in the main body, where relates more to "litigation duration". Think about changing the term in the main body, or the title. I prefer to "duration", since efficiency is a much complicated term.<br>Though the data is available in the web, you may probably tell me the link of the data source in the footnote, so I can be more convinced. I have searched two websites you provided. There are many data within, and I can't identify which data you are using.<br>Apart from what mentioned above, I think it is a valuable article.	(1)On the Appropriateness of the Term "Efficiency" in the Title<br>As you pointed out, the main focus of the study lies in "litigation duration" rather than "efficiency." To avoid any potential misinterpretation, I have revised the title to replace "efficiency" with "duration." Additionally, all relevant terms in the main text have been updated to ensure consistency with the revised title.<br>(2)On the Clarity of Data Sources<br>The data that support the findings of this study are openly available in Data and procedures at http://dx.doi.org/10.5281/zenodo.14238241<br>If you need the original data you can find it by using the following database addresses and filters:<br>Charitable Giving Data: This was obtained from the CSMAR database. After accessing the database, search using the Chinese terms “non-operating income” and “non-operating expenditure.” After retrieving the raw data, manual sorting of donation items is required to calculate the annual donation amounts for listed companies.<br>Litigation Data: This was retrieved from the CNRDS database. Use the Chinese term “Listed Company Litigation and Arbitration Statistics” in the search bar to locate the relevant data.<br>Control Variable Data: This was sourced from the CSMAR database. Simply search for the names of the specific control variables in the search bar to retrieve the data.<br>If you have any questions regarding the data, please feel free to contact me. I am committed to ensuring the accuracy, transparency, and traceability of the data used in this study, as I recognize the importance of data validation in the review process.<br>(3)On Concerns About Duplicate Submissions<br>Thank you for raising this critical point. I assure you that this study is entirely original and has not been published in any other journal nor submitted elsewhere. I will maintain close communication with the editorial office to ensure full compliance with academic ethics throughout the publication process. Furthermore, I solemnly commit that if	

		this article is accepted , it will not be published or disseminated in any form in other journals, including those in China. I strictly adhere to principles of academic integrity and will ensure the uniqueness and exclusivity of this article.<br>Thank you again for your detailed feedback and support. Please do not hesitate to reach out if further clarification or information is required.	
	**Reviewer-4**		
1	While the paper addresses endogenous issues using instrumental variables, it would be beneficial to delve into other possible sources, including reverse causality or omitted variable bias.	I have addressed the potential endogeneity issues arising from reverse causality and omitted variable bias in the manuscript. To mitigate these concerns, I employed an instrumental variable (IV) approach to reduce estimation errors caused by potential endogeneity. The results obtained using the IV method confirm that the impact of charitable donations on litigation duration remains consistent with the baseline regression results, further supporting the robustness of the findings.<br>In addition, I have elaborated on the relevance and exogeneity of the instrument used—the average donation levels classified by province, industry, and ownership type. This instrument was selected based on its ability to satisfy both relevance (correlation with corporate charitable donations) and exogeneity (uncorrelated with the error term in the litigation duration model). These additions are detailed in the revised manuscript to enhance the theoretical and empirical foundation of the study.<br>If you have further suggestions or require additional analyses, I am happy to make the necessary adjustments.	See pages 24-25 of the Manuscript.
2	The core contribution is the reputation mechanism as a pathway for charitable donations to impact litigation duration, but its exact function needs deeper revelation. Intermediate variable analysis is suggested to clarify how donations affect litigation through	(1)Clarifying how charitable donations influence litigation through reputation:<br>In the revised manuscript, I have included additional empirical results to explain how charitable donations impact litigation efficiency via corporate reputation. Specifically, I employed Poisson regression to capture the mapping relationship between charitable donations and corporate reputation and then used OLS regression to estimate the effect of corporate reputation on litigation duration. These supplementary results and discussions have been incorporated into the	See pages 26-27 and 8-9 of the Manuscript.

	reputation. Additionally, other potential mechanisms like political connections and social networks should not be overlooked; their relationships with variables should be addressed even if not expanded upon.	revised manuscript, providing a clearer demonstration of the causal chain: "Charitable Donations → Corporate Reputation → Litigation Duration."<br>(2)Addressing the relationship between the reputation mechanism and alternative mechanisms, such as political connections and social networks:<br>I acknowledge the importance of considering alternative mechanisms, such as political connections and social networks, in shaping the relationship between charitable donations and litigation duration. To address this, I have expanded the literature review to include discussions on these potential mechanisms and their theoretical relevance. While the current analysis focuses on the reputation mechanism, I have also accounted for these factors in the empirical framework by controlling for related variables (e.g., Soe, Government, Law, Shrcr1, and Separation) in the regression models. These controls aim to minimize potential estimation bias stemming from alternative mechanisms.<br>I hope these revisions address your valuable suggestions. Please let me know if further clarification or additional analyses are required.	
3	The paper explores the influence of case types and regions on the donation-litigation relationship but lacks discussion on the theoretical mechanisms behind these heterogeneous effects. Its analysis of regional variations in donation, allocation, and litigation duration is somewhat cursory.	In the revised manuscript, I strengthened the discussion on the mechanism of the impact of case type and regional heterogeneity on the relationship between donation and litigation duration. As for case type, the reason for the heterogeneity is that the judicial department has a large difference in discretion. For civil and arbitration cases, the courts often have a large discretion, while in administrative and criminal cases, other public power agencies such as government agencies and procuratorates are involved. Therefore, the courts have little room for discretion over the litigation cycle. Therefore, even if enterprises actively donate, they cannot affect the litigation cycle of their cases. In terms of regional heterogeneity, I have supplemented both the discussion and	See pages 28-31 of the Manuscript.

		empirical part. I supplemented the regional heterogeneity analysis of the sub-sample of listed companies as plaintiffs, and added relevant regression results and explanations in the manuscript.	
4	The mention of the Civil Code's maximum trial duration for civil suits on page 4 contrasts with later comparisons involving administrative, criminal, and arbitration procedures for listed companies. China's legislation sets different trial duration rules for various procedures, which should be distinguished.	I distinguished the longest trial periods for different types of cases and added an appendix to list them. The main text uses the trial period of civil litigation (the type of case with the most litigation involving listed companies) to illustrate that the court has a certain degree of discretion over the litigation period of the company. There are similar provisions for administrative, criminal and arbitration cases. It is worth noting that in our classification discussion, the essence is to focus on the differences in litigation cycles between listed companies, and the longest trial cycle faced by the court is correspondingly given. The difference in the longest trial cycle between case types does not affect the analysis of our grouped empirical results and corresponding conclusions. In order to avoid the main text being too lengthy, the longest trial period for each type is not listed in the main text. According to your suggestion, we have classified and listed the longest trial periods of the first instance of civil, administrative, criminal and arbitration cases in the appendix of the article.	See pages 42-45 of the Manuscript.
5	The conclusion and implications section should further propose specific policy recommendations, such as encouraging corporate donations and improving the judicial system for litigation efficiency, particularly addressing whether current trial duration rules need revision.	Based on your suggestions, I put forward policy recommendations from both the corporate and judicial perspectives and listed them in the conclusion section. And I think based on my research, there is no need to revise the current trial duration rules for two reasons:First, the principle of legal stability requires cautious consideration before making changes to trial duration rules. Second, the findings of this study do not aim to assess the reasonableness of these rules. Instead, I focus on how judicial authorities, operating under the existing framework, are influenced by corporate behaviors and respond with flexible actions.	See pages 37-38 of the Manuscript.

I would like to specifically address Reviewer 3’s concerns regarding the potential existence of a Chinese version of this manuscript. I can unequivocally confirm that this study has not been

published in any Chinese journal or other media. I fully understand and respect the journal's academic integrity and copyright policies, and I assure you that this manuscript, if accepted, will not be submitted, translated, or published elsewhere, including on any Chinese platform or in any other format.

The manuscript represents entirely original research, and its theoretical framework, methodology, and empirical findings were developed specifically for this submission. If any additional documentation or verification is needed, I am more than happy to provide further clarification or supporting evidence to address any concerns.

Additionally, I have made other adjustments to improve the clarity and readability of the paper, including, but not limited to, revising the format of the paper by plos one and standardising the number of decimal places as required . I have attached the Revised Manuscript with Track Changes for your review.

Thank you again for your insightful feedback and support. I hope the revised version aligns with the journal's standards and expectations and will be favorably considered for publication.

Sincerely,
Feng Zhu
