# Supplementary material for: How charitable giving affects litigation duration? Empirical evidence from China
Source: PLoS One. 2024 Dec 30;19(12):e0316180. doi: 10.1371/journal.pone.0316180 (PMC11684597; doi:10.1371/journal.pone.0316180)
Supplement: Attachment — Submitted filename: Review Comments.docx [file pone.0316180.s002.docx]

This is an intriguing study that elucidates how charitable donations, by influencing corporate reputation, alter case processing order and judicial resource allocation, thereby providing a novel theoretical foundation for understanding the complexity and externalities of judicial activities and enriching legal and economic research. The study employs quantitative methods and adequately explains its methodology and data, leading to highly credible conclusions. However, there is several areas for improvement:

1.While the paper addresses endogenous issues using instrumental variables, it would be beneficial to delve into other possible sources, including reverse causality or omitted variable bias.

2.The core contribution is the reputation mechanism as a pathway for charitable donations to impact litigation duration, but its exact function needs deeper revelation. Intermediate variable analysis is suggested to clarify how donations affect litigation through reputation. Additionally, other potential mechanisms like political connections and social networks should not be overlooked; their relationships with variables should be addressed even if not expanded upon.

3.The paper explores the influence of case types and regions on the donation-litigation relationship but lacks discussion on the theoretical mechanisms behind these heterogeneous effects. Its analysis of regional variations in donation, allocation, and litigation duration is somewhat cursory.

4.The mention of the Civil Code's maximum trial duration for civil suits on page 4 contrasts with later comparisons involving administrative, criminal, and arbitration procedures for listed companies. China's legislation sets different trial duration rules for various procedures, which should be distinguished.

5.The conclusion and implications section should further propose specific policy recommendations, such as encouraging corporate donations and improving the judicial system for litigation efficiency, particularly addressing whether current trial duration rules need revision.
