# Supplementary material for: How charitable giving affects litigation duration? Empirical evidence from China
Source: PLoS One. 2024 Dec 30;19(12):e0316180. doi: 10.1371/journal.pone.0316180 (PMC11684597; doi:10.1371/journal.pone.0316180)
Supplement: Attachment — Submitted filename: Pl.docx [file pone.0316180.s004.docx]

Thank you for the author's revision, which included following the reviewers’ suggestions and demonstrating good data management in the manuscript. I strongly recommend that the manuscript be published after minor corrections. Please, author, add a section “Future Research Directions” after the Conclusions section. Additionally, I suggest citing the newly published research articles related to the author’s study, which I have provided below.

The requirements for filing a consumer public interest litigation in China.

<https://doi.org/10.1504/MEJM.2024.135152>

Digital Technology Application and Enterprise Competitiveness: The Mediating Role of ESG Performance and Green Technology Innovation. Environ Dev Sustain 2023, doi:10.1007/s10668-023-03979-3.

The Role of Technology in the Digital Economy’s Sustainable Development of Hainan Free Trade Port and Genetic Testing: Cloud Computing and Digital Law. <https://doi.org/10.3390/su16146025>

Management Economic Systems and Governance to Reduce Potential Risks in Digital Silk Road Investments: Legal Cooperation between Hainan Free Trade Port and Ethiopia. <https://doi.org/10.3390/systems12080305>
